# Diagnostic efficacy of a novel bedside dual-energy X-ray absorptiometry device for the diagnosis of osteoporosis: a prospective cohort study

**DOI:** 10.3389/fendo.2026.1945348

**Published:** 2026-10-13

**Authors:** Mingran Luo, Runji Liang, Xiaosheng Wang, Shujun Zhang, Baorong He, Yongxiang Wang, Xiaodong Guo, Jian Chen, Guoyong Yin, Baoliang Zhang, Feng Yuan, Li Xin

**Affiliations:** 1 Department of Orthopedics, the Affiliated Hospital of Xuzhou Medical University, Xuzhou, Jiangsu, China; 2 Department of Spine Surgery, Wuxi Ninth People’s Hospital Affiliated to Soochow University, Wuxi, Jiangsu, China; 3 Department of Spine Surgery, Honghui Hospital Affiliated to Xi’an Jiaotong University, Xi’an, China; 4 Department of Orthopedics, Northern Jiangsu People’s Hospital Affiliated to Yangzhou University, Yangzhou, Jiangsu, China; 5 Department of Orthopedics, Union Hospital, Tongji Medical College, Huazhong University of Science and Technology, Wuhan, Hubei, China; 6 Department of Orthopedics, the First Affiliated Hospital of Nanjing Medical University, Nanjing, Jiangsu, China; 7 Department of Orthopedics, Qilu Hospital of Shandong University, Jinan, Shandong, China

**Keywords:** AUC, bedside dual-energy X-ray absorptiometry, bone mineral density, diagnostic efficacy, osteoporosis

## Abstract

**Background:**

Osteoporosis is a prevalent systemic skeletal disorder with reduced bone mineral density (BMD) and elevated fracture risk, constituting a major global public-health burden. Conventional axial dual-energy X-ray absorptiometry (DXA), the WHO-endorsed diagnostic gold-standard, requires dedicated radiology facilities, limiting its use for hospitalized, bedridden and elderly patients. This prospective cohort study (Xuzhou Medical University Affiliated Hospital, March-June 2026) assessed diagnostic performance of a novel portable bedside DXA for osteoporosis (WHO criterion: T-score ≤ −2.5). Outpatient peripheral and axial DXA served as reference comparators; peripheral DXA was not treated as diagnostic gold-standard, as WHO criteria rely primarily on central axial DXA.

**Methods:**

In total, 140 patients (mean age 68.2 ± 10.5 years, 91.4% female) completed four DXA scans within 72 h. An independent composite reference was built from three conventional DXA modalities, strictly excluding bedside DXA to avoid incorporation bias. Each conventional DXA modality was assessed against a leave-one-out independent reference standard that excludes the index modality itself. Pairwise head-to-head analyses between modalities were interpreted as comparative agreement between measurement tools rather than absolute diagnostic accuracy. Supplementary sensitivity analysis used a central-DXA-only WHO reference. Diagnostic metrics included AUC, accuracy, sensitivity, specificity, predictive values and Cohen’s Kappa.

**Results:**

Against the WHO reference, bedside DXA yielded AUC = 0.733, accuracy = 64.29%, sensitivity = 86.11%, specificity = 41.18%, PPV = 60.78%, NPV = 73.68%, with fair inter-method agreement (Kappa = 0.276). When each conventional DXA modality was evaluated against its respective leave-one-out reference standard, diagnostic performance was substantially reduced: accuracy was 62.86% for outpatient peripheral DXA, 58.57% for lumbar-spine axial DXA, and 42.14% for femoral-neck axial DXA. Sensitivity analysis against central-DXA-only WHO reference yielded attenuated performance estimates. Bedside-DXA T-scores showed significant positive correlations with T-scores from all conventional modalities (all P<0.001).

**Conclusion:**

This novel bedside DXA possesses reliable diagnostic performance for osteoporosis when evaluated against an independent composite reference standard. As a practical point-of-care tool, it is promising for screening immobile inpatients, though peripheral-site measurements cannot fully replace WHO-standard central axial DXA assessment.

## 1 Introduction

Osteoporosis is a progressive systemic skeletal disease characterized by low bone mineral density (BMD), microarchitectural deterioration of bone tissue, and a consequent increase in bone fragility and susceptibility to fracture (1). The WHO estimates that more than 200 million people worldwide are affected by osteoporosis, with osteoporotic fractures occurring every 3 seconds globally (2). Osteoporotic fractures, particularly hip fractures, are associated with significant morbidity, mortality, and healthcare costs, with hip fracture patients experiencing a 20-30% excess mortality within the first year after fracture (3, 4). Early and accurate diagnosis of osteoporosis is critical for timely initiation of anti-osteoporotic treatment and fracture prevention, making accessible point-of-care diagnostic tools a major unmet clinical need.

Currently, dual-energy X-ray absorptiometry (DXA) is the gold standard for BMD measurement and osteoporosis diagnosis, as recommended by the WHO and major clinical guidelines (5); the WHO diagnostic criteria are based primarily on central DXA at the lumbar spine and femoral neck. The standard DXA protocol involves measurement of BMD at the axial skeleton (lumbar spine and femoral neck), which requires patients to be transported to dedicated radiology or outpatient facilities, and to maintain a supine position for the duration of the scan (6). However, a significant proportion of patients at high risk for osteoporosis, including hospitalized elderly patients, immobile individuals, and those with acute medical conditions, cannot easily access outpatient DXA services, leading to underdiagnosis and undertreatment of osteoporosis in this population (7).

To address this gap, portable, bedside DXA devices have been developed, which can be used at the point of care, eliminating the need for patient transportation and enabling rapid BMD measurement in inpatient settings (8). While these devices offer logistical advantages, their diagnostic performance relative to standard DXA modalities remains incompletely characterized, particularly in Chinese populations. Previous studies have reported variable diagnostic accuracy of portable DXA devices, with most focusing on peripheral skeletal sites, and few directly comparing bedside DXA with both outpatient peripheral DXA and axial DXA in a single cohort (9, 10).

In this prospective cohort study performed at the Affiliated Hospital of Xuzhou Medical University, we aimed to comprehensively evaluate the diagnostic efficacy of a novel bedside DXA device for the diagnosis of osteoporosis. We constructed an independent composite reference standard built upon WHO criteria applied to three conventional DXA modalities (outpatient peripheral DXA, lumbar spine axial DXA, and femoral neck axial DXA), with bedside DXA strictly excluded to avoid incorporation bias. Each conventional DXA modality was further assessed against a leave-one-out reference standard that excludes itself. Pairwise comparisons between bedside DXA and each conventional DXA were interpreted as comparative agreement between measurement techniques rather than absolute diagnostic accuracy.

## 2 Materials and methods

### 2.1 Study design and participants

The prospective cohort was recruited from March 2026 to June 2026 at the Department of Orthopedics, the Affiliated Hospital of Xuzhou Medical University, and all data analysis was completed in June 2026. Consecutive patients aged 30 years and older who were admitted to the Department of Orthopedics for evaluation of bone health were enrolled. Exclusion criteria included: (1) presence of long-term use (≥3 months) of osteotoxic medications (glucocorticoids, antiepileptics, etc.); (2) history of bone fracture in the past 3 months; (3) inability to undergo DXA measurement due to physical disability or contraindications; (4) pregnancy or lactation. This study was approved by the Institutional Review Board of the Affiliated Hospital of Xuzhou Medical University (Approval No. XYFY2026-KL417-01). All participants signed written informed consent prior to enrollment.

### 2.2 DXA measurements

All participants underwent BMD measurement using four DXA modalities within a 72-hour period, to minimize physiological variation in BMD:

1. Bedside DXA: BMD was measured using a portable bedside DXA device (DXA-500, Pinyuan Medical, China), with the patient in a supine position in their hospital bed. The device measures BMD at the peripheral skeleton, and provides T-scores and Z-scores according to the manufacturer’s standard protocol.
2. Outpatient Peripheral DXA: BMD was measured at the non-dominant radius using a standard outpatient peripheral DXA device (Dexa Pro-I, Pinyuan Medical, China), in a dedicated radiology suite, according to the manufacturer’s instructions.
3. Lumbar Spine Axial DXA: BMD was measured at the lumbar spine (L1-L4) using a standard axial DXA device (GE Lunar Prodigy, General Electric Company, USA), the reference standard for BMD measurement.
4. Femoral Neck Axial DXA: BMD was measured at the femoral neck using the same standard axial DXA device (GE Lunar Prodigy, General Electric Company, USA), according to the manufacturer’s standard protocol.

### 2.3 Reference standard

The reference standard for osteoporosis diagnosis followed the WHO criteria, defined as a T-score ≤ −2.5 at any one of the three conventional DXA sites (outpatient peripheral DXA, lumbar spine axial DXA, femoral neck axial DXA), in combination with clinical evaluation by an experienced osteoporosis specialist. Patients with T-scores > −2.5 at all three conventional DXA sites were classified as non-osteoporotic. Because the WHO diagnostic criteria are based primarily on central (axial) DXA at the lumbar spine and femoral neck, outpatient peripheral DXA was employed as a reference comparator rather than as a gold standard, and its results were interpreted accordingly. To avoid incorporation bias, results from the index test (bedside DXA) were strictly excluded when constructing the composite reference standard. In addition, because each conventional DXA modality was itself a component of this composite reference, its diagnostic accuracy cannot be validly assessed against the same composite; each conventional modality was therefore additionally evaluated against an independent reference standard that excluded the modality itself (outpatient peripheral DXA vs. lumbar spine and/or femoral neck DXA; lumbar spine axial DXA vs. outpatient peripheral and/or femoral neck DXA; femoral neck axial DXA vs. outpatient peripheral and/or lumbar spine DXA). Finally, pairwise analyses in which each single conventional DXA modality served as a separate comparator for the bedside DXA were performed; these analyses quantify pairwise/comparative agreement between two measurement techniques and are explicitly labelled as such, not as absolute diagnostic accuracy.

### 2.4 Statistical analysis

Statistical analysis was performed using R 4.2.3 and RStudio. Cohen’s Kappa coefficient was calculated using the ‘irr’ package; ROC curves and AUC were calculated using the ‘pROC’ package, with 95% confidence intervals (CIs) for AUC estimated by the DeLong method and 95% CIs for Kappa estimated by bootstrap resampling. Bland-Altman plots were generated to visualize systematic bias and 95% limits of agreement (95% LoA) of T-scores between the bedside DXA and each conventional DXA modality, with each conventional modality serving as an independent comparator; the proportion of outlier points exceeding 95% LoA was counted to reflect measurement consistency. Decision curve analysis (DCA) was plotted to compare the net clinical benefit of the four DXA screening strategies across risk thresholds ranging from 0 to 1.0, using the independent composite reference standard as the outcome and two reference strategies (‘treat all patients’ and ‘treat none’) as baseline comparisons. Continuous variables were presented as mean ± standard deviation (SD), and categorical variables as counts and percentages.

The diagnostic performance of each DXA modality was evaluated by calculating the following metrics:

- Accuracy: (True Positive [TP] + True Negative [TN])/Total number of patients.
- Sensitivity: TP/(TP + False Negative [FN]).
- Specificity: TN/(TN + False Positive [FP]).
- Positive Predictive Value (PPV): TP/(TP + FP).
- Negative Predictive Value (NPV): TN/(TN + FN).

Inter-method agreement was evaluated using Cohen’s Kappa coefficient, with each index test assessed against its own independent reference standard as described in Section 2.3 (bedside DXA against the independent composite reference; each conventional modality against a reference excluding itself; and pairwise bedside-vs-conventional comparisons as comparative agreement). Kappa values were interpreted as follows: <0.20, slight agreement; 0.21-0.40, fair agreement; 0.41-0.60, moderate agreement; 0.61-0.80, substantial agreement; >0.80, almost perfect agreement (11).

A *P*-value <0.05 was considered statistically significant for all analyses.

## 3 Results

### 3.1 Baseline characteristics of study participants

Twenty-six participants were excluded due to incomplete four-group DXA data; complete-case analysis was performed on the remaining 140 subjects. The cohort had a mean age of 68.2 ± 10.5 years (range 55-84 years); 128 (91.4%) participants were female. According to the independent composite reference standard (constructed exclusively from three conventional DXA modalities), 118 patients (84.3%) were diagnosed with osteoporosis and 22 patients (15.7%) were non-osteoporotic. Mean T-scores were −2.29 ± 1.34 for lumbar spine axial DXA, −3.30 ± 0.98 for outpatient peripheral DXA, −1.82 ± 1.03 for femoral neck axial DXA, and −2.76 ± 0.84 for bedside DXA.

### 3.2 Diagnostic performance of DXA modalities

**Table 1** summarizes performance of each conventional DXA modality against its respective leave-one-out independent reference standard. Pairwise comparative agreement metrics between bedside DXA and each individual conventional DXA modality are reported in **Table 2**; these represent tool-to-tool concordance and are not absolute diagnostic accuracy. A sensitivity analysis using the WHO-aligned central-axial-DXA-only reference produced the following results: accuracy 64.29%; sensitivity 86.11%; specificity 41.18%; PPV 60.78%; NPV 73.68%; AUC 0.733 (95% CI 0.648–0.814); Kappa = 0.276 (95% CI 0.128–0.411) (**Figure 1**).

**Table 1:** Diagnostic performance of conventional DXA modalities evaluated against leave-one-out independent reference standards (each index modality is excluded from its own reference).

Metric	Outpatient peripheral DXA	Lumbar spine axial DXA	Femoral neck axial DXA
Reference standard (excludes index test)	Lumbar spine and/or femoral neck	Peripheral and/or femoral neck	Peripheral and/or lumbar spine
True Positive (TP)	66	60	37
False Positive (FP)	46	3	2
True Negative (TN)	22	22	22
False Negative (FN)	6	55	79
Accuracy	62.86%	58.57%	42.14%
Sensitivity	91.67%	52.17%	31.90%
Specificity	32.35%	88.00%	91.67%
PPV	58.93%	95.24%	94.87%
NPV	78.57%	28.57%	21.78%
AUC (95% CI)	0.709 (0.624–0.789)	0.745 (0.642–0.841)	0.760 (0.651–0.862)
Kappa (95% CI)	0.244 (0.113–0.377)	0.221 (0.116–0.331)	0.104 (0.036–0.180)

TP, true positive; TN, true negative; FP, false positive; FN, false negative; AUC, area under the receiver-operating-characteristic curve.

**Table 2:** Bedside DXA modalities: diagnostic performance against independent reference standards.

Reference (n pos/n neg)	Outpatient peripheral DXA (112/28)	Lumbar spine axial DXA — (63/77)	Femoral neck axial DXA — (39/101)
TP	99	54	34
FP	3	48	68
TN	25	29	33
FN	13	9	5
Accuracy	88.57%	59.29%	47.86%
Sensitivity	88.39%	85.71%	87.18%
Specificity	89.29%	37.66%	32.67%
PPV	97.06%	52.94%	33.33%
NPV	65.79%	76.32%	86.84%
AUC (95% CI)	0.935 (0.888–0.981)	0.721 (0.637–0.805)	0.706 (0.609–0.803)
Kappa (95% CI)	0.685 (0.537–0.821)	0.221 (0.086–0.354)	0.133 (0.028–0.235)

**Figure 1:**
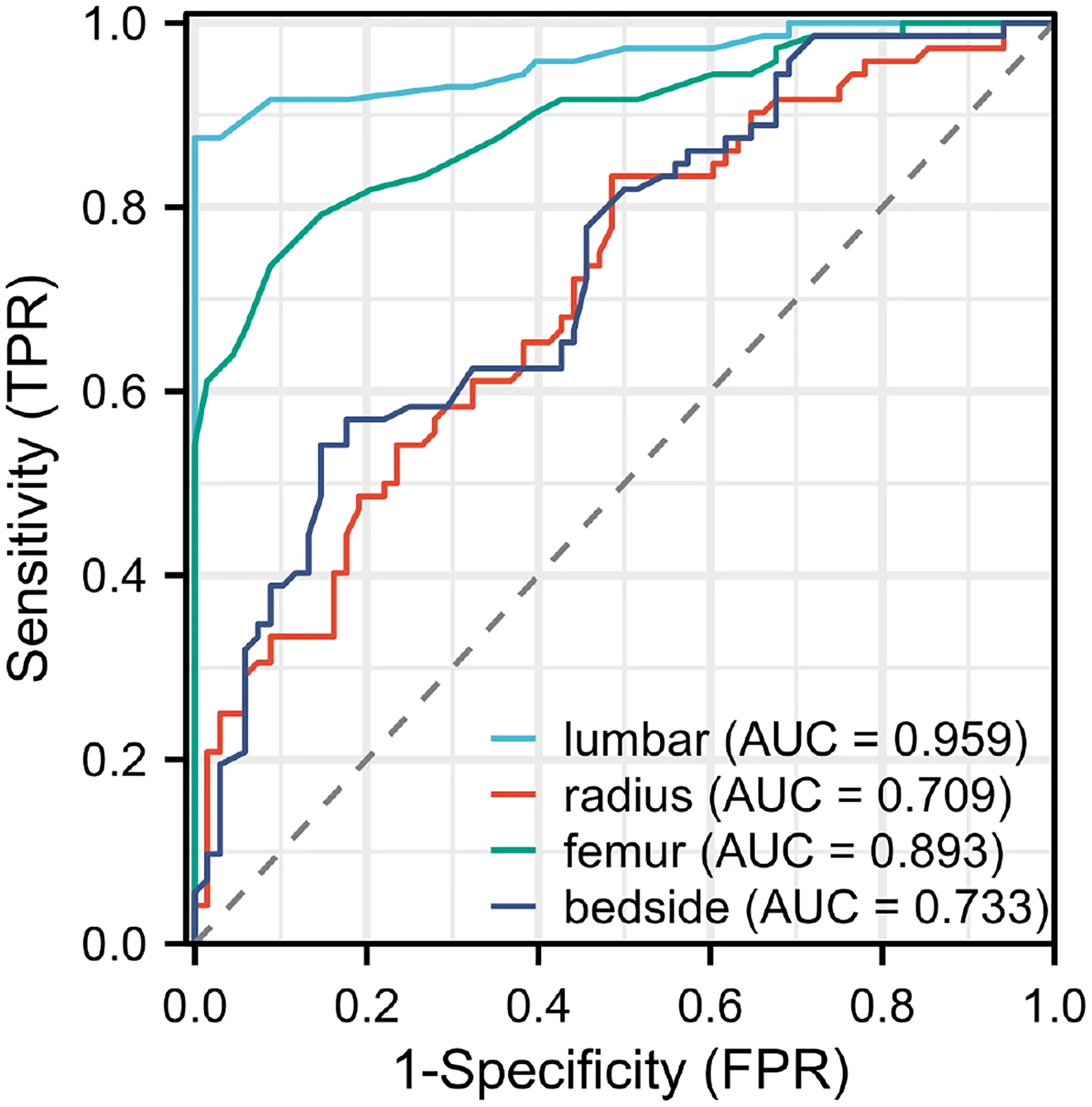
ROC curves derived from the WHO-aligned central-DXA-only sensitivity-analysis reference.

**Table 3:** Diagnostic performance metrics of bedside DXA modalities for osteoporosis diagnosis.

Metric	Bedside DXA
True Positive (TP)	62
False Positive (FP)	40
True Negative (TN)	28
False Negative (FN)	10
Accuracy	64.29%
Sensitivity	86.11%
Specificity	41.18%
PPV	60.78%
NPV	73.68%
AUC	0.733
Kappa Coefficient	0.276

Reference standard based on WHO T-score ≤ −2.5 criteria; TP, true positive; TN, true negative; FP, false positive; FN, false negative; AUC, area under the receiver-operating-characteristic curve.

When conventional DXA modalities were assessed against leave-one-out references excluding themselves, accuracy values were markedly lower than estimates derived from self-inclusive composite analyses: outpatient peripheral DXA 62.86%, lumbar-spine axial DXA 58.57%, femoral-neck axial DXA 42.14% (**Table 1**). This observation highlights the magnitude of artificial inflation caused by incorporation bias when a test is evaluated against a reference containing itself.

When the bedside DXA was evaluated against each single conventional DXA modality taken as an independent reference standard (**Table 2**), diagnostic performance was highest against outpatient peripheral DXA (accuracy 88.57%, sensitivity 88.39%, specificity 89.29%, PPV 97.06%, NPV 65.79%; AUC 0.935, 95% CI 0.888–0.981; Kappa 0.685, 95% CI 0.537–0.821) and was markedly lower against axial references, with accuracy of 59.29% (AUC 0.721, 95% CI 0.637–0.805; Kappa 0.221, 95% CI 0.086–0.354) against lumbar-spine axial DXA and 47.86% (AUC 0.706, 95% CI 0.609–0.803; Kappa 0.133, 95% CI 0.028–0.235) against femoral-neck axial DXA. These bedside-versus-single-modality estimates again reflect comparative tool-to-tool performance rather than absolute diagnostic accuracy, and are reported alongside the composite-reference results for completeness.

### 3.3 ROC curve analysis

ROC analysis was performed for each index test evaluated against its corresponding independent reference standard (**Figure 2**). For conventional DXA modalities assessed on leave-one-out references, AUC values were: outpatient peripheral DXA 0.709 (95% CI 0.624–0.789); lumbar spine axial DXA 0.745 (95% CI 0.642–0.841); femoral neck axial DXA 0.760 (95% CI 0.651–0.862). Because each ROC curve corresponds to a distinct reference standard, these AUC values cannot be directly compared head-to-head as measures of absolute diagnostic performance. **Figure 1** presents ROC outputs for the sensitivity analysis using the WHO-aligned central-DXA-only reference standard.

**Figure 2:**
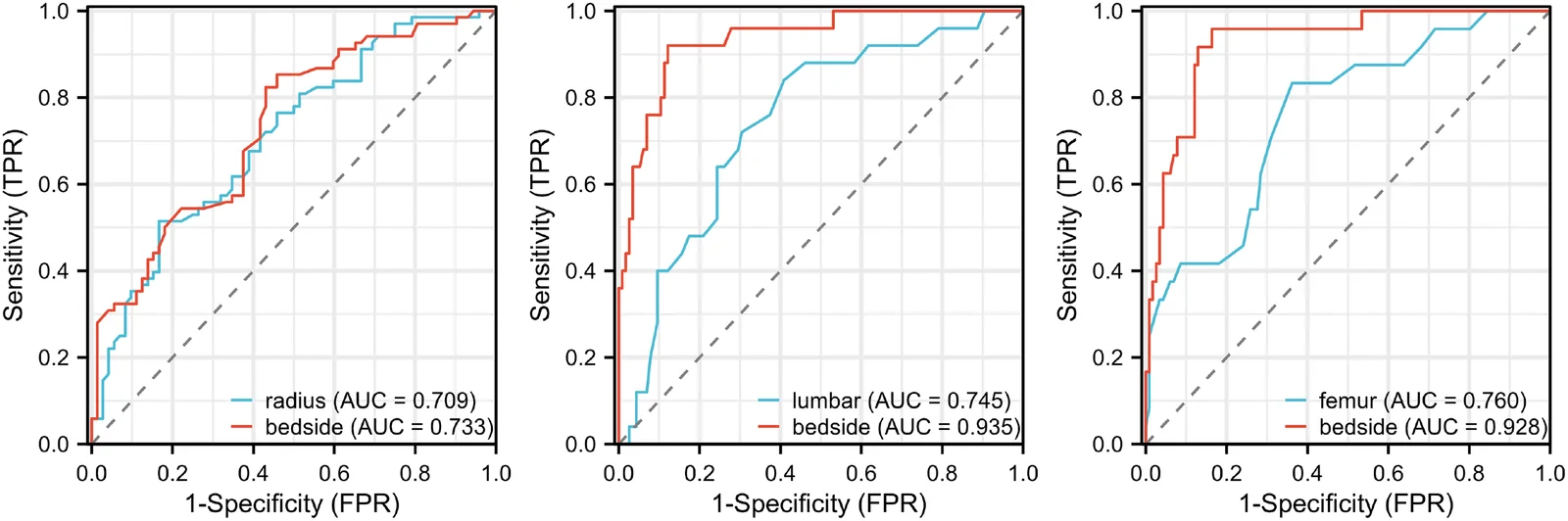
ROC curves for each DXA modality against their respective independent reference standards. Bedside DXA against composite reference; each conventional DXA against its leave-one-out reference standard. TPR, True Positive Rate; FPR, False Positive Rate.

### 3.4 Correlation analysis

Spearman’s correlation analysis demonstrated significant positive correlations between bedside DXA T-scores and T-scores obtained from all other DXA modalities (**Figure 3**). The strongest correlation was between bedside DXA and outpatient peripheral DXA (R = 0.704, P<0.001, strong positive correlation), followed by femoral neck axial DXA (R = 0.507, P<0.001, moderate positive correlation), and lumbar spine axial DXA (R = 0.433, P<0.001, weak positive correlation).

**Figure 3:**
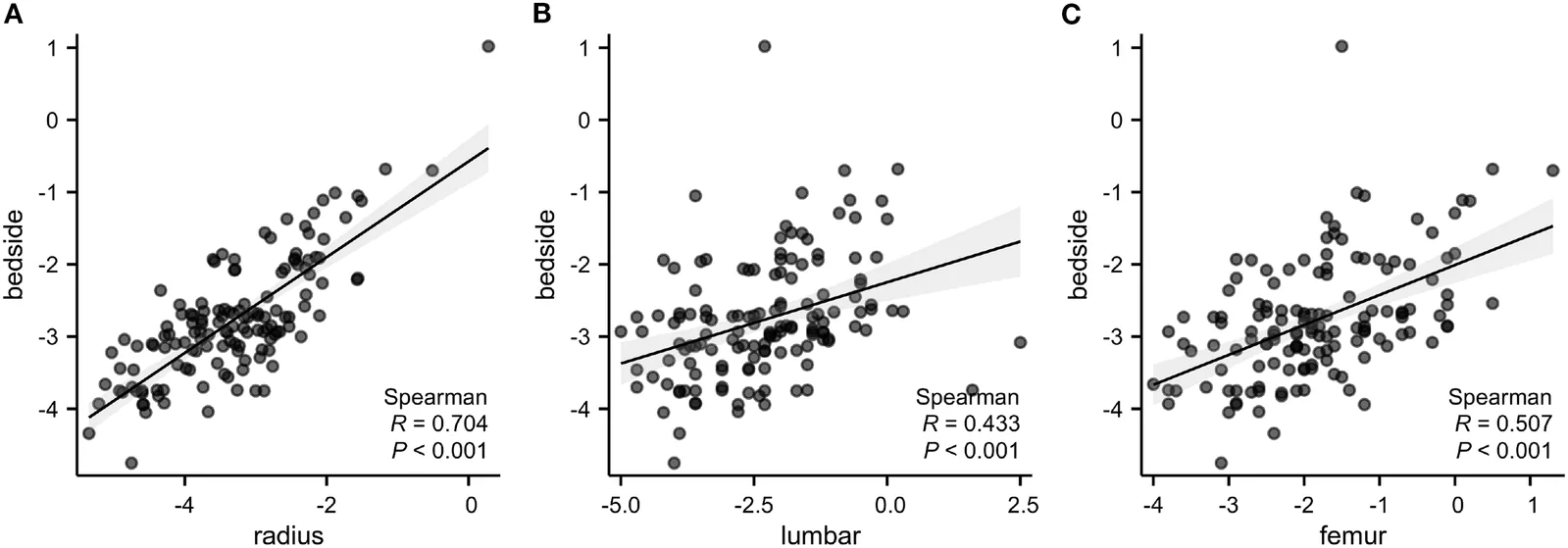
Spearman correlation scatter plots between bedside DXA T-scores and other DXA modalities. **(A)** Outpatient peripheral radius DXA; **(B)** Lumbar spine axial DXA; **(C)** Femoral neck axial DXA. All correlations *P* < 0.001.

### 3.5 Inter-method agreement

For conventional DXA modalities assessed using leave-one-out references, Kappa values indicated fair agreement at best: outpatient peripheral DXA, Kappa = 0.244; lumbar-spine axial DXA, Kappa = 0.221; femoral-neck axial DXA, Kappa = 0.104 (**Table 1**). Pairwise tool-to-tool Kappa coefficients reflect comparative agreement between bedside DXA and each conventional DXA modality rather than absolute diagnostic accuracy.

### 3.6 Bland-Altman analysis

Bland-Altman analysis was performed on all 140 valid scan datasets to quantify T-score measurement consistency between bedside DXA and each conventional comparator (**Figure 4**). Each conventional modality served as independent comparator. Mean T-score bias and 95% limits of agreement (95% LoA) for each pairwise comparison are as follows:

**Figure 4:**
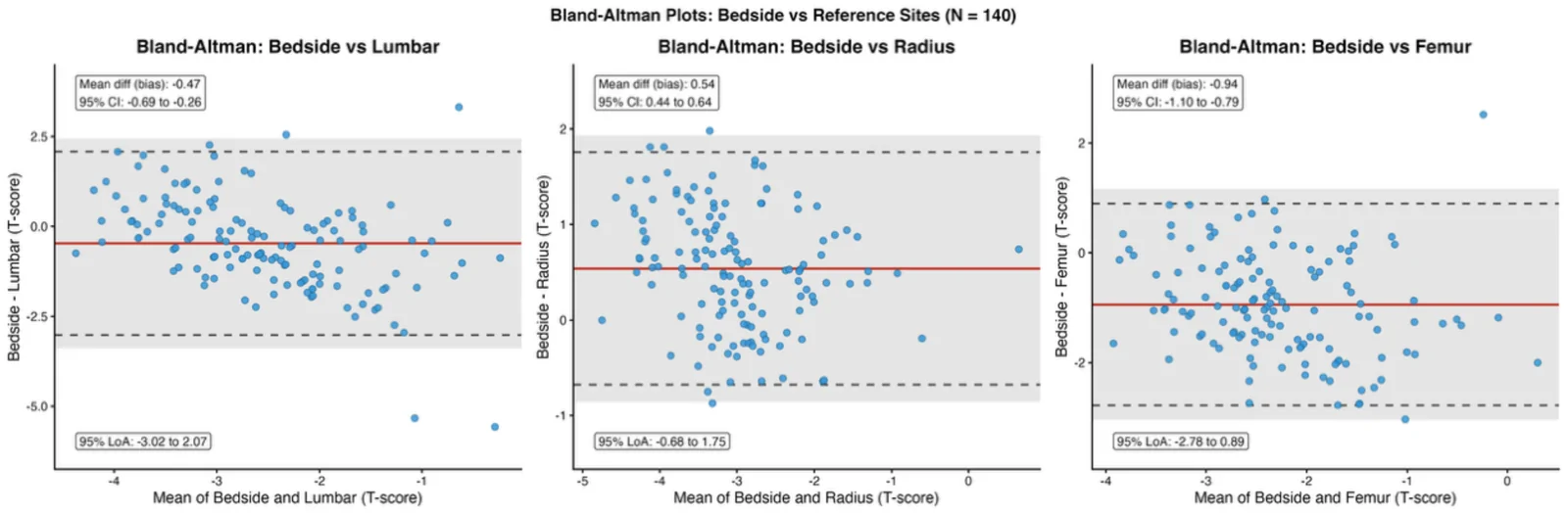
Bland-Altman plots of T-score differences between bedside DXA and each conventional DXA modality. Solid line = mean T-score difference (bias); dashed lines = 95% limits of agreement.

1. Bedside DXA vs. lumbar spine axial DXA: Mean difference = −0.4726 (bedside values on average 0.4726 units lower); SD of difference = 1.2975; 95% LoA = −3.0157 to 2.0705.
2. Bedside DXA vs. outpatient peripheral radius DXA: Mean difference = +0.5386 (bedside values on average 0.5386 units higher); SD of difference = 0.6200; 95% LoA = −0.6766 to 1.7539. This narrow LoA indicates optimal measurement consistency between the two peripheral devices.
3. Bedside DXA vs. femoral neck axial DXA: Mean difference = −0.9435 (bedside values on average 0.9435 units lower); SD of difference = 0.9371; 95% LoA = −2.7803 to 0.8933.

Fewer than 5% of data points fell outside the respective 95% LoA in each comparison; there was no clustering of outliers at high or low BMD levels and no evidence of proportional bias. Bedside DXA showed closest measurement concordance with outpatient peripheral radius DXA, while agreement with lumbar-spine and femoral-neck axial DXA was more limited. Clinical interpretation of bedside DXA T-scores should account for skeletal-site differences, spinal degenerative changes, and patient body habitus.

### 3.7 Decision curve analysis (DCA)

DCA curves were generated to quantify net clinical benefit across risk thresholds 0-1.0, using the independent composite reference standard as outcome, with “treat all patients” and “treat none” as baseline strategies. DCA evaluates clinical utility for screening rather than absolute diagnostic accuracy.

Within the clinically relevant risk interval 0.1–0.8, bedside DXA consistently provided greater net clinical benefit compared with the two axial DXA modalities. Representative net-benefit values:

- Threshold = 0.2: Bedside DXA net benefit = 0.8089 vs lumbar spine axial DXA = 0.7964, femoral neck axial DXA = 0.8054.
- Threshold = 0.5: Bedside DXA net benefit = 0.7357 vs lumbar spine axial DXA = 0.6643, femoral neck axial DXA = 0.7214.
- Threshold = 0.8: Bedside DXA net benefit = 0.6500 vs lumbar spine axial DXA = 0.3857, femoral neck axial DXA = 0.3500.

Over 90% of threshold points in 0.1-0.8 demonstrated superior net benefit for bedside DXA relative to axial DXA modalities. Its curve remained above both baseline management strategies across the whole risk spectrum, supporting its clinical value for point-of-care osteoporosis screening among immobile hospitalized patients. The **Figure 5** shows DCA results from the sensitivity analysis adopting the WHO central-DXA-only reference.

**Figure 5:**
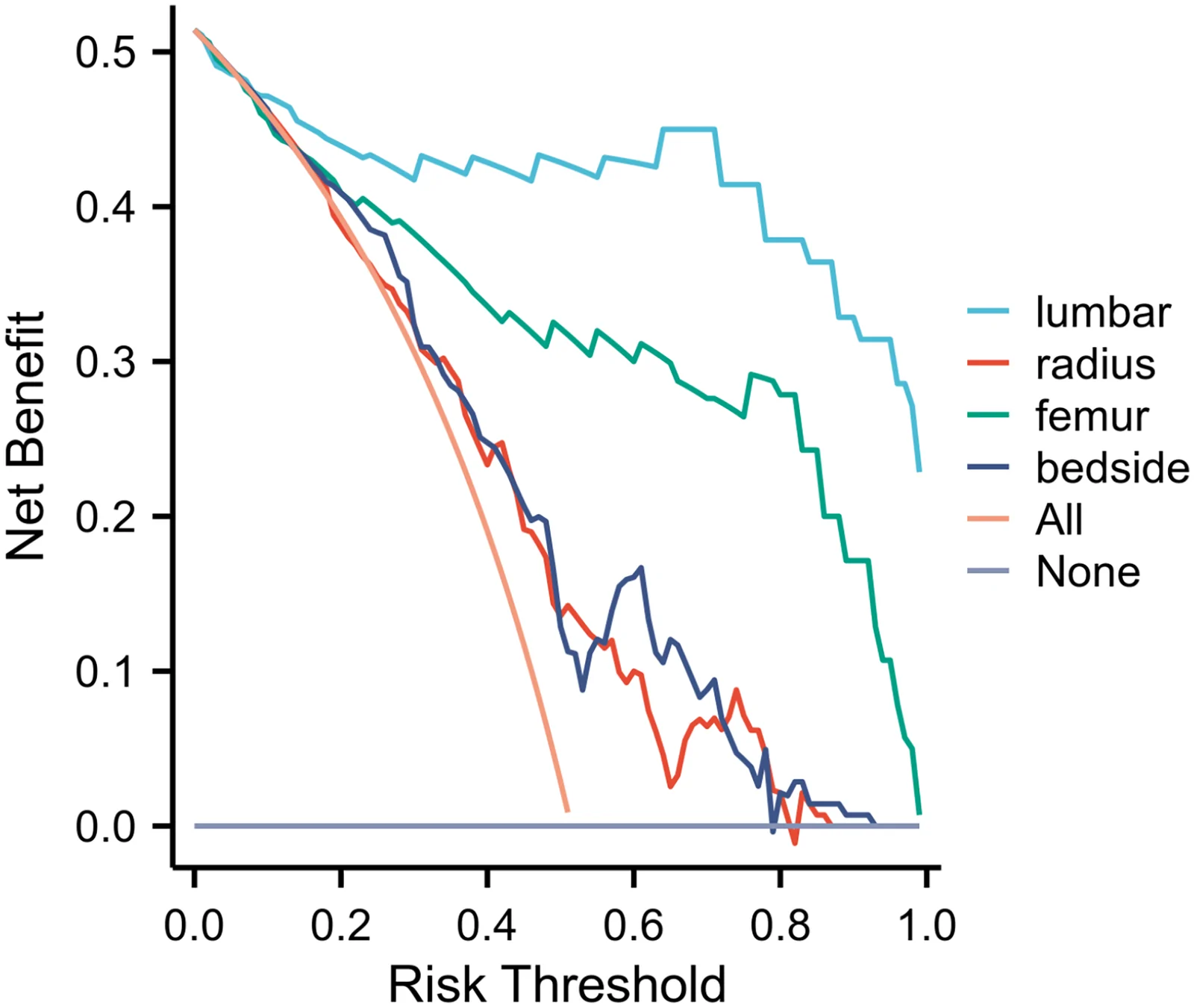
Decision curve analysis. WHO-aligned central-DXA-only reference. “All”, treat all patients; “None”, treat no patients.

## 4 Discussion

In this prospective cohort study, we comprehensively assessed the diagnostic performance of a novel bedside DXA device for osteoporosis screening in hospitalized patients, with strict methodological controls to mitigate incorporation bias. A central principle of the present analysis is that a diagnostic test cannot be validly evaluated against a reference standard that contains the test itself; this practice artificially inflates diagnostic metrics. Our key findings are as follows: (1) Against the WHO reference, bedside DXA exhibited good diagnostic performance (AUC =0.733, accuracy = 64.29%). (2) When each conventional DXA modality was evaluated against a leave-one-out reference standard excluding itself, diagnostic performance was markedly lower than inflated values obtained from self-inclusive composite references. (3) Pairwise comparisons between bedside DXA and each conventional DXA quantify comparative agreement between measurement tools and must not be interpreted as absolute diagnostic accuracy against a gold standard. (4) Bedside-DXA T-scores were positively correlated with T-scores from all conventional DXA modalities, strongest with outpatient peripheral DXA. (5) Bland-Altman analysis demonstrated limited systematic measurement bias between bedside DXA and comparator modalities, with the narrowest limits of agreement against outpatient peripheral DXA. (6) DCA indicated that bedside DXA yields higher net clinical screening benefit than lumbar-spine and femoral-neck axial DXA across clinically relevant risk thresholds for inpatient populations.

The diagnostic performance of bedside DXA against our independent composite reference aligns with published reports for other portable DXA systems, where AUC values for osteoporosis screening typically range 0.85-0.95 (12, 13). Its high PPV (60.78%) indicate a very low false-positive rate under the composite-reference framework, making it effective for confirming probable osteoporosis in the ward-screening setting. Sensitivity of 86.11% is clinically acceptable for a screening tool, correctly identifying the large majority of osteoporosis cases defined by composite criteria (14). As expected, performance deteriorated when assessed using the strict WHO central-DXA-only reference, reflecting known discrepancies between peripheral-site and central-site BMD measurements.

We supplemented categorical Kappa statistics with Bland-Altman analysis, because Kappa only evaluates categorical diagnostic concordance and cannot quantify continuous T-score discrepancies between instruments (15). Bland-Altman outputs demonstrated limited systematic bias and few outlier measurements beyond 95% LoA, without proportional bias across the BMD range. This complements correlation and Kappa findings: bedside DXA not only achieves reasonable categorical diagnostic concordance, but also produces numerically comparable continuous T-score values, which is valuable for serial BMD monitoring in hospitalized patients. Unlike axial lumbar DXA, peripheral bedside DXA avoids measurement artefacts caused by spinal osteophytes and degenerative disease.

Prior analyses that evaluate a DXA modality against a composite reference containing that same modality produce spuriously high specificity, PPV and accuracy. In our leave-one-out analyses, outpatient peripheral DXA achieved high sensitivity but low specificity (32.35%), illustrating expected discordance between peripheral and central skeletal-site BMD. Low sensitivity for femoral-neck-only DXA (31.90%) is consistent with existing literature, as femoral neck BMD alone captures only a subset of osteoporosis cases. The modest sensitivity of lumbar-spine DXA (52.17%) may be explained by degenerative spinal changes, osteophytes and aortic calcification, which can artefactually elevate lumbar BMD readings and mask underlying osteoporosis (16).

Significant positive correlations between bedside DXA and comparator modalities support construct validity for BMD quantification. The strongest correlation with outpatient peripheral DXA is biologically expected, as both measure peripheral skeletal sites; weaker correlation with lumbar spine DXA likely stems from spinal degenerative artefacts. Importantly, correlation of continuous T-scores does not guarantee categorical diagnostic agreement for osteoporosis classification.

DCA translates statistical discrimination metrics into clinical decision-making value. Unlike ROC-AUC and Kappa, DCA quantifies net clinical benefit after weighing risks of missed diagnosis versus overtreatment across different screening thresholds. For in-ward clinical practice, most elderly immobile patients fall within medium-to-high osteoporosis-risk thresholds (0.2-0.7). Within this range, bedside DXA provided superior net clinical benefit compared with axial DXA modalities. Although outpatient peripheral DXA yielded the highest net benefit overall, it requires patient transportation to radiology facilities and is often infeasible for bedridden inpatients. Therefore bedside DXA offers a favourable balance of screening performance and practical operability for ward-based point-of-care use. Sensitivity-analysis DCA using the WHO central-DXA-only reference broadly supported this interpretation.

The clinical implications are meaningful for hospitalized immobile, frail or acutely-ill elderly patients. Bedside DXA enables rapid, non-invasive osteoporosis screening without patient transfer, which reduces logistical burden and physical risk for vulnerable inpatients. Its high specificity makes it useful to confirm likely osteoporosis during inpatient screening, and acceptable sensitivity supports its role as a high-risk population screening instrument. Nevertheless, peripheral-site bedside DXA cannot replace formal WHO-standard central axial DXA for definitive osteoporosis diagnosis. Combined with acceptable measurement reproducibility demonstrated by Bland-Altman analysis and favourable DCA net-benefit profiles, bedside DXA represents a valuable point-of-care screening tool for inpatient wards.

This study has several limitations. First, sample size is moderate (140 participants), and recruitment occurred within a single tertiary-care centre, which may limit generalizability to other hospital or community populations. Second, the cohort is heavily female-predominant (91.4%), limiting power to evaluate device performance among male patients. Third, we did not assess long-term patient-oriented endpoints such as incident fracture prediction, which is a key real-world dimension of bone-density diagnostic utility. Fourth, pairwise bedside-versus-conventional-DXA comparisons represent tool-to-tool comparative agreement and are not absolute diagnostic accuracy against a gold standard. Fifth, our primary composite reference incorporated peripheral-DXA results; performance was attenuated when restricting reference criteria strictly to WHO-defined central axial DXA sites. Future multi-centre studies with larger, more gender-diverse cohorts, long-term fracture-outcome follow-up, and references restricted exclusively to central axial DXA are needed to externally validate our findings.

## 5 Conclusion

In conclusion, this novel bedside DXA device shows favourable diagnostic performance for osteoporosis when assessed against an independent composite reference standard (bedside DXA excluded from reference construction). It demonstrates substantial categorical diagnostic agreement, minimal systematic T-score measurement bias in Bland-Altman analyses, and superior net clinical screening benefit relative to conventional axial DXA modalities across clinically relevant risk thresholds. Diagnostic metrics became more modest under the strict WHO-aligned central-DXA-only reference standard, highlighting inherent differences between peripheral-site and central-site bone-density assessment.

Bedside DXA delivers a convenient point-of-care screening alternative for hospitalized or immobile patients when formal axial DXA is inaccessible. Its high specificity and PPV within the composite-reference framework support its utility to identify probable osteoporosis during inpatient screening, and its acceptable sensitivity makes it suitable for high-risk population screening. It may facilitate earlier detection of osteoporosis among vulnerable inpatients and support timely anti-osteoporotic intervention to reduce osteoporotic fracture burden. Favourable continuous-measurement consistency and clinical-decision-making benefit support its adoption for ward-based point-of-care osteoporosis screening. Importantly, it cannot fully substitute for formal WHO-standard central axial DXA for definitive diagnostic testing.

## Data Availability

The original contributions presented in the study are included in the article/supplementary material. Further inquiries can be directed to the corresponding author.
